# Bethlem myopathy: A novel homozygous variant of c.385C>T (p.Arg129Cys) in the COL6A2 gene

**DOI:** 10.1002/ccr3.9306

**Published:** 2024-08-12

**Authors:** Maryam Kachuei, Kiana Orangi, Aynaz Mohammadi, Mohammad Mohammadi, Marzieh Mojbafan

**Affiliations:** ^1^ Firoozabadi Clinical Research Development Unit, Department of Pediatrics Iran University of Medical Sciences Tehran Iran; ^2^ School of Medicine Iran University of Medical Sciences Tehran Iran; ^3^ Department of Medical Genetics, School of Medicine Iran University of Medical Sciences Tehran Iran

**Keywords:** Bethlem myopathy, case report, congenital disorder, genetic testing, rare disease

## Abstract

**Key Clinical Message:**

This case highlights the challenges in diagnosing Bethlem myopathy, the need for a high index of suspicion, and the importance of recognizing the diverse clinical presentations of this rare condition. Enhanced understanding can aid in early diagnosis and tailored management.

**Abstract:**

Bethlem myopathy (BM), a rare collagen VI‐related myopathy, is characterized by progressive muscle weakness and contractures, typically affecting the proximal muscles and joints. This case report presents a 15‐year‐old girl from Tehran, Iran, with a 5‐year history of severe limb pain and progressive weakness. Born to consanguineous parents, the patient displayed delayed walking milestones and significant hypotonia, leading to a waddling gait and lumbar hyperlordosis. Neurological examination revealed marked proximal lower limb weakness, a positive Gowers' sign, and absent myotatic reflexes. Elevated creatine phosphokinase (CPK) levels and electromyography (EMG) results indicated myopathy, while nerve conduction studies showed no neuropathy. Genetic testing revealed a novel homozygous variant of c.385C>T (p.Arg129Cys) in the COL6A2 gene, classified as a variant of uncertain significance (VUS) per American College of Medical Genetics and Genomics (ACMG) guidelines due to its rarity and specific phenotype association. Differential diagnosis is essential to distinguish it from other neuromuscular conditions. Management primarily focuses on symptom relief and enhancing patients' quality of life. This case highlights the challenges in diagnosing BM, the need for a high index of suspicion, and the importance of recognizing the diverse clinical presentations of this rare condition. Enhanced understanding can aid in early diagnosis and tailored management.

## 1 INTRODUCTION

Bethlem myopathy (BM), initially documented by Bethlem and Wijngaarden,^1^ is an uncommon condition with an estimated prevalence of 0.77 cases per 100,000 individuals in Northern England.^2^ This condition falls within the category of congenital muscular dystrophies, specifically referred to as “collagen VI‐related myopathies.”^3^ Collagen VI, a nonfibrillar collagen, plays a crucial role in various connective tissues, including tendons, ligaments, muscles, skin, cornea, cartilage, bone tissue, and periosteum. It is a heterotrimeric protein comprised of three distinct alpha chains and is involved in essential cellular processes like adhesion, migration, and apoptosis through interactions with cell‐surface molecules.^4^

BM typically manifests with proximal muscle weakness and contractures affecting the fingers, wrists, elbows, and ankles as its primary symptoms. However, the presentation of BM varies considerably, with some cases showing the absence of either contractures or muscular weakness.^5^ Its slowly progressive nature often leads to misdiagnosis.

Diagnosis relies on clinical assessment and laboratory evaluations, with genetic testing serving as a confirmatory tool.^6^ Recent studies have highlighted the utility of magnetic resonance imaging (MRI) in diagnosing collagen VI‐related myopathies, particularly through the identification of characteristic MRI signs like the “sandwich” sign in the vastus lateralis (VL) and the “target” sign in the rectus femoris (RF).^7^ These MRI findings have demonstrated a positive predictive value of 69% for BM.^8^

Treatment approaches for BM are tailored to individual symptoms, but commonly involve physical therapy. In select cases, surgical interventions may be necessary to address joint contractures.^6^ Currently, no cure exists for BM, and treatment primarily focuses on symptom management and enhancing the quality of life for affected individuals.^9^

## 2 CASE HISTORY/EXAMINATION

We received a referral for a 15‐year‐old girl in Tehran, Iran, from a low socio‐economic background with third‐degree cousin parents. About 5 years ago, she began experiencing severe limb pain, particularly in her feet, leading to restlessness, and impairment. At the same time, progressive limb weakness appeared, now completely disabling her. Seeking local medical advice initially attributed the pain to normal growth discomfort, but as daily life difficulties persisted due to intensifying pain and weakness, they approached our center.

Despite an uneventful birth, her walking milestone was slightly delayed at 14 months. Until age 10, her development was normal. At 10, severe pain and weakness emerged, resulting in significant hypotonia, a waddling gait, and lumbar hyperlordosis. Additionally, the patient's legs are significantly undersized relative to her age, and the mother reports progressive muscle atrophy in the patient's legs. However, the patient does not exhibit distal joint laxity or any specific contractures. The patient's facial appearance is normal, with no evident deformities. Furthermore, the patient does not have any skin lesions or corneal problem. Neurological exams revealed proximal lower limb weakness, hindering stair climbing. Muscle strength tests rated her 2 out of 5, with a positive Gowers' sign. Absent myotatic reflexes and normal sensory results were noted.

The patient's parents lack neuromuscular issues, but two aunts and one uncle (the father's siblings) began to experience gradual muscle weakness at age 30, but without much pain. The patient's 8‐year‐old half‐sister, who shares the same mother, has had severe pain and weakness since age 4. Genetic tests confirmed she has BM.

## 3 METHODS (DIFFERENTIAL DIAGNOSIS, INVESTIGATIONS, AND TREATMENT)

The differential diagnosis of BM involves distinguishing it from other conditions with similar clinical features, such as Emery–Dreifuss muscular dystrophy (EDMD), early‐onset limb‐girdle muscular dystrophy (LGMD), Ehlers–Danlos syndrome (EDS), and Ullrich congenital muscular dystrophy (UCMD). EDMD can be distinguished by the presence of cardiac electrical abnormalities and dilated cardiomyopathy, conditions that are not present in BM. In our case, there are no cardiac problems present. Certain forms of LGMD, particularly those presenting with proximal muscle weakness, can be distinguished from BM using immunohistochemistry and serum CPK levels, with BM typically presenting with mildly elevated CK levels, as seen in our case. EDS should be considered in patients with joint laxity and characteristic skin hyperlaxity, which can help differentiate it from BM. Muscle biopsy and immunostaining techniques targeting collagen VI and other markers typically yield normal results in BM, whereas UCMD shows decreased or absent collagen VI immunostaining. Given the patient's symptoms, which were less severe than those seen in UCMD and progressed more slowly, BM was deemed a more suitable diagnosis.

Clinical examination focused on patient history, symptom progression, developmental milestones, and family history, with particular attention to muscle strength, gait, and neurological assessment. Laboratory tests revealed elevated creatine phosphokinase (CPK) levels at 413 U/L. Electromyography (EMG) indicated myopathy, while nerve conduction velocity (NCV) tests showed no signs of neuropathy. Whole‐exome sequencing identified a novel homozygous variant of c.385C>T (p.Arg129Cys) in the COL6A2 gene, classified as a variant of uncertain significance (VUS) according to ACMG guidelines. Although a muscle MRI was not conducted, a brain MRI showed no pathology. Treatment focused on symptomatic management, including physical therapy to improve muscle strength and mobility, and pain management with analgesics and supportive care. Genetic counseling was provided to the patient's family to discuss the genetic findings and potential hereditary patterns.

## 4 CONCLUSION AND RESULTS (OUTCOME AND FOLLOW‐UP)

The definitive diagnosis of BM in this case was established through genetic testing, which identified a novel homozygous variant of c.385C>T (p.Arg129Cys) in the COL6A2 gene. This finding underscores the significance of genetic testing in cases where clinical presentation is consistent with BM but lacks definitive diagnostic features through other modalities.

The management plan primarily focuses on symptomatic treatment and supportive care. Physical therapy has been initiated to address muscle weakness and maintain joint mobility. The patient's progress is closely monitored with periodic assessments to evaluate the effectiveness of the treatment and adjust the care plan as needed. Additionally, the patient and her family have received genetic counseling to better understand the implications of the diagnosis and the potential for recurrence in future pregnancies.

The follow‐up strategy includes regular clinical evaluations to monitor disease progression, assess muscle strength, and detect any emerging complications.

## 5 DISCUSSION

BM, a rare condition, was initially characterized in 1976 by Bethlem and Wijngaarden in a study involving 28 patients from diverse families.^1^ It is noteworthy that BM is predominantly inherited in an autosomal dominant manner, and it falls within the category of congenital muscular dystrophies.^9^ Clinically, it is characterized by a gradual onset of muscle weakness, which primarily affects the lower extremities and, to a lesser extent, the upper limbs. This weakness progressively worsens over time,^6^, ^9^ as observed in our case as well. Several other distinctive features of BM include the development of contractures in the hands and feet. These contractures result in a “tightness” or restricted range of motion in the fingers and Achilles tendons.^6^

While respiratory muscles are affected to some extent, it's essential to emphasize that severe respiratory complications are rare in BM, typically manifesting in late adulthood.^6^ The disorder is also associated with skin abnormalities, such as keloid formation, “cigarette paper scarring” (atrophic scarring), velvety soft skin, and follicular hyperkeratosis.^9^

The genetic basis of BM lies in mutations within the COL6A1, COL6A2, or COL6A3 genes.^6^ While most cases follow an autosomal dominant inheritance pattern, there are rare instances of autosomal recessive transmission,^6^ as seen in this case.

In this study, we report a novel homozygous variant of c.385C>T (p.Arg129Cys) in the COL6A2 gene. Common mutations in the COL6A1, COL6A2, and COL6A3 genes typically present with phenotypic features such as muscle weakness and contractures.^5^ Our case, involving a novel mutation, provides an opportunity to explore deviations in the clinical phenotype associated with this new genotype.

Our patient exhibited classical symptoms of BM, including proximal muscle weakness and spine deformity. However, she did not exhibit some typical features associated with BM, such as distal joint laxity, specific contractures, or facial dysmorphisms. This variation in clinical presentation suggests that the novel c.385C>T (p.Arg129Cys) mutation might influence the phenotype differently compared to more common mutations.

An important diagnostic tool in BM is muscle imaging, which exhibits a highly specific pattern of muscle involvement with a sensitivity of around 90%.^8^, ^10^ A notable characteristic is the peripheral involvement of the vasti muscles and the anterocentral involvement of the RF. This pattern can be easily recognized and greatly aids in achieving a definitive genetic diagnosis.^8^, ^10^, ^11^

In a comprehensive discussion of BM, it is also essential to address its differential diagnosis to avoid confusion with other conditions that share some clinical features. For instance, conditions like EDMD^12^ and specific variants of early‐onset LGMD present muscular weakness and contractures similar to BM.^13^ However, EDMD can be differentiated by the presence of cardiac electrical abnormalities and the eventual development of dilated cardiomyopathy. Muscle MRI findings can be useful as well, showing predominantly posterior compartment involvement in EDMD, as opposed to the anterior muscle involvement seen in BM.^14^

Certain forms of LGMD may resemble BM, particularly when the primary clinical signs are related to proximal muscle weakness. In such cases, immunohistochemistry using specific antibodies and serum CPK levels play a crucial role in distinguishing the two conditions. BM typically presents with mildly elevated CK levels, whereas most LGMD variants exhibit significantly elevated CK levels, as seen in conditions like sarcoglycanopathies and dysferlinopathies.^13^

It's also essential to consider the possibility of EDS when assessing patients with joint laxity, especially in teenagers or adults. The presence of characteristic skin hyperlaxity can help differentiate EDS from BM. Though BM patients may frequently exhibit features like keloids and hyperkeratosis, these are not essential for a clinical diagnosis of BM.^15^

In terms of laboratory testing, muscle biopsy, and immunostaining techniques with antibodies targeting collagen VI, as well as markers such as sarcoglycans, caveolin, dysferlin, α‐dystroglycan, and merosin, typically yield normal results in BM patients. This stands in stark contrast to UCMD, where these techniques reveal a decreased or total absence of collagen VI immunostaining. This difference in immunostaining serves as a significant distinguishing factor between the two phenotypes.^4^

## 6 CONCLUSION AND RESULTS

To summarize, we documented a novel homozygous variant, c.385C>T (p.Arg129Cys), in the COL6A2 gene for BM, a rare congenital muscular dystrophy exhibiting distinctive clinical features. It bears similarities to conditions such as EDMD, specific LGMD variants, and laminopathy. Precise diagnosis and differentiation are pivotal for tailored management. Essential tools for this include genetic testing, muscle imaging, and clinical assessments. A deeper comprehension of these conditions significantly improves the care provided to affected individuals.

## AUTHOR CONTRIBUTIONS

**Maryam Kachuei:** Conceptualization; writing – review and editing. **Kiana Orangi:** Writing – original draft; writing – review and editing. **Aynaz Mohammadi:** Project administration; supervision; writing – original draft; writing – review and editing. **Mohammad Mohammadi:** Writing – original draft; writing – review and editing. **Marzieh Mojbafan:** Writing – review and editing.

## FUNDING INFORMATION

No funding was received.

## CONFLICT OF INTEREST STATEMENT

The authors declare that they have no known competing financial interests or personal relationships that could have appeared to influence the work reported in this paper.

## CONSENT

Written informed consent was obtained from the patient to publish this report in accordance with the journal's patient consent policy.

## Data Availability

The data are available for sharing.
